# The Harveian Oration

**Published:** 1894-10-27

**Authors:** 


### THE HARVEIAN ORATION.

The oration delivered last Thursday by Dr. Lauder Brunton was full of suggestiveness. After a fitting tribute to the importance of Harvey's work, he pointed to the wide and unending influence of that simple idea which he had expressed in the words: "I began to think whether there might not be motion, as it were, in a circle." On this hinged the whole of our knowledge of the physiology of the circulation, and on physiological knowledge must now hang the treatment we apply to the varied forms of heart disease. Probably the most interesting part of the oration, especially as being explanatory of much that is important in regard to treatment, was his exposition of the effects of muscular exertion in producing strain upon the heart, the first effect being to increase the resistance, owing to the contracting muscle opposing the passage of blood, the next to lessen it, from the arteries dilating, and the third to increase the frequency of the heart's action, so as to replace the blood flowing away through the dilated arteries. All these must act in harmony, and in cases where the arteries are rigid, and do not dilate in response to the demand, the initial strain is not relieved, and dangerous results may follow. In all cases, however, if over-exertion be persisted in, the cavities of the heart become considerably dilated, and much mischief is thus often done to growing boys by the prolonged effort of paper-chases and enforced races.

The rationale of the treatment of most cases of heart disease is then to increase the power of the heart; to lessen the resistance it has to overcome; and in some cases to aid in the removal of the water which has accumulated in the form of dropsy. The importance of physiological considerations in laying down a course of treatment are sufficiently obvious. A regimen suitable to one case may be fatal to another; sudden and violent exertion may raise the blood pressure and so lead to intense cardiac pain, or to stoppage of the heart and instant death; while more gentle exercises by opening the arterioles and increasing the circulation through the muscles may lessen pressure and relieve the heart.

In regard to treatment by drugs Dr. Lauder Brunton makes the very important suggestion that the action of many eliminative medicines may influence the economy less by what they eliminate than by the new chemical compounds which result in what remains. The idea is gaining ground that, just as in the case of the ductless glands, so in the excretory glands, new products may be poured into the blood stream, and, for example, the benefit of diaphoresis may arise not from mere excretion of the sweat, but from products poured into the blood by the increased activity of the sudoriparous glands. The constituents of the perspiration may then be merely the chips and refuse left after the constructive activity of these glands; their real secretion may be something poured into the blood, something essential to health; and the excretory effects of both purgatives and diaphoretics may be but a small item in their restorative influence. All of which is in full accord with clinical observation.

